# Association between gut microbiota, plasma metabolites, and ovarian cancer: A Mendelian randomization study

**DOI:** 10.1097/MD.0000000000040479

**Published:** 2024-11-08

**Authors:** Yu Wang, Shanxiang Gao, Yangyu Liu, Yongai Li, Hui Yao, Yan Han, Xinyue Liu

**Affiliations:** a The Pathology Department of Changzhi People’s Hospital Affiliated to Changzhi Medical College, Changzhi, China; b Dalian Medical University, Dalian, China; c The Pathology Department of Shanxi Provincial People’ Hospital, Shanxi Medical University, Taiyuan, China; d Medical Imaging Center of Changzhi People’s Hospital Affiliated to Changzhi Medical College, Changzhi, China; e The Gynecology of Changzhi People’s Hospital Affiliated to Changzhi Medical College, Changzhi, China; f The Gynecology Department of Shanxi Provincial People’ Hospital, Shanxi Medical University, Taiyuan, China.

**Keywords:** gut microbiota, mediation analysis, Mendelian randomization, ovarian cancer, plasma metabolites

## Abstract

Numerous studies have demonstrated a correlation between alterations in gut microbiota (GM) and levels of body metabolites in ovarian cancer (OC). However, the specific causal relationships underlying these associations remain unclear. This study utilized summary statistics of GM from the MiBioGen consortium, along with an unprecedented dataset comprising 1091 blood metabolites and 309 metabolite ratios from the UK Biobank, in conjunction with OC data from the FinnGen Consortium R9 release. We conducted bidirectional Mendelian randomization (MR) analyses to investigate the causal relationships between GM and OC. Additionally, a two-step MR approach was employed to identify potential mediating metabolites. Our analysis revealed significant associations between 6 specific microbiota taxa and OC. Furthermore, we identified several plasma metabolites that act as mediators of the association between GM and OC. In the two-step MR analysis, we observed a negative correlation between 4-methoxyphenol sulfate and pregnenetriol disulfate levels with OC. The genus *Lachnospiraceae UCG008* potentially increases the risk of OC by decreasing 4-methoxyphenol sulfate levels, while the genus *Howardella* may elevate the risk of OC by reducing pregnenetriol disulfate levels, with mediation proportions of 22.35% and 4.23%, respectively. Additionally, levels of dilinoleoyl-GPE (18:2/18:2) and N-acetylkynurenine (2) were positively correlated with OC. The inhibitory effect of the genus *Ruminococcus 1* on OC may be mediated through 1,2-dilinoleoyl-GPE (18:2/18:2) and N-acetylkynurenine (2), with mediation proportions of 10.15% and 11.32%, respectively. Our findings highlight the complex relationship among GM, plasma metabolites, and OC. The identified associations and mediation effects offer valuable insights into potential therapeutic approaches targeting GM for the management of OC.

## 1. Introduction

Ovarian cancer (OC) is among the most prevalent malignant tumors in the field of gynecology, and it is the primary cause of death among cancers affecting the female reproductive system. The prognosis for OC is unfavorable, with a 5-year survival rate of approximately 47%.^[1]^ In recent years, the potential connection between the microbiota and the development of cancer has emerged as a topic of increasing interest and debate. Among the different regions of the body, the human gut microbiota (GM) is particularly noteworthy as a vital constituent of the human microbiome, displaying the greatest bacterial richness and diversity.^[2]^ Dysbiosis in the GM seems to contribute to the development of cancer by disrupting the physiological balance of intestinal epithelial cells. A representative microbiota has been identified in women with OC: a unique set of viruses, bacteria, fungi, and parasites have been identified in a pan-pathogen array.^[3]^ Substantial efforts have been devoted to elucidating the pathophysiological mechanisms underlying carcinogenesis driven by microbial factors. Well-established factors, such as the tumor-promoting microenvironment, epithelial barrier dysfunction, altered metabolic processes, and immune dysregulation, have been linked to specific bacteria within dysbiotic microbial communities.^[4]^ Research has demonstrated that an imbalance in the GM can impact the levels of human metabolites, thereby influencing tumor occurrence and development.^[5–10]^ Given that patients diagnosed with OC frequently experience gastrointestinal symptoms like abdominal pain, bloating, indigestion, constipation, and early satiety, exploring the potential link between alterations in the GM and OC carcinogenesis is indeed intriguing. Nevertheless, the research on the complex interactions between GM, associated metabolites, and OC has a rather limited scope. Therefore, there is a need for comprehensive exploration to understand the complex mechanisms by which changes in GM and related metabolites impact OC development and treatment pathways, as well as to identify potential metabolites that can be utilized for early diagnosis and clinical therapeutic targets.

Mendelian randomization (MR) is a widely accepted method that employs genetic variants as instrumental variables (IVs) to control for potential confounding factors, thereby avoiding reverse causation bias and enabling more reliable causal inferences between exposure and clinical outcomes.^[11]^ To identify specific metabolite levels that mediate the relationship between GM composition and the onset or progression of OC, we conducted a bidirectional MR study and performed two-step mediation analyses using summary statistics from the largest and most up-to-date genome-wide association studies (GWAS) of the GM, 1400 plasma metabolites, and OC.

In conclusion, this study aims to bridge the knowledge gap between GM, plasma metabolites, and the pathogenesis of OC. By utilizing advanced genetic techniques and conducting a comprehensive analysis of plasma metabolites, this research aims to elucidate potential therapeutic targets and gain a deeper understanding of the intricate etiology of OC. These findings could be valuable for prevention, reducing morbidity, managing recurrence, and minimizing side effects through interventions targeting specific GM taxa and plasma metabolites.

## 2. Methods

### 2.1. Study design

**Figure 1.**
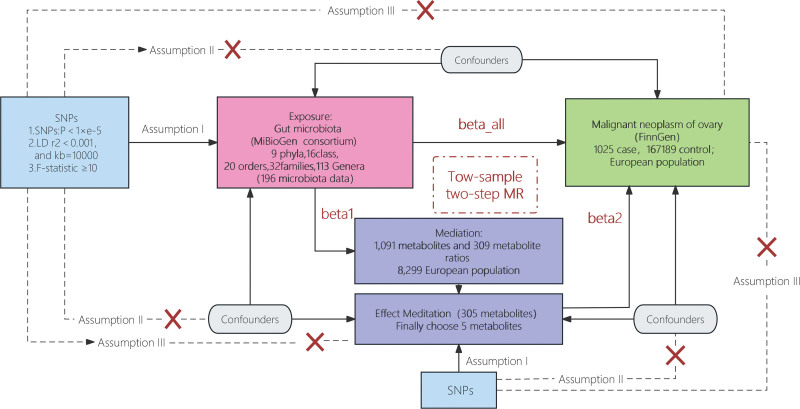
Mendelian randomization analysis flow chart.

The research hypotheses and flow chart are shown in Figure 1. We employed a two-sample MR analysis^[12]^ to evaluate the causal relationship between GM and OC. Additionally, to further investigate the mediating role of plasma metabolites, we conducted a two-step MR analysis to assess the effects of potential mediators.^[13]^ Specifically, in an MR analysis, the genetic variants employed as IVs, referred to as single nucleotide polymorphisms (SNPs), should adhere to 3 primary assumptions^[14]^: (I) there should be a robust association between the IVs and the exposure; (II) the IVs should be free from confounding; (III) the IVs should exclusively impact the outcome through the exposure. To ensure the integrity of the analysis, genetic data related to GM, plasma metabolites, and OC were extracted from separate GWAS datasets, thereby ensuring the elimination of sample overlap.

### 2.2. Data sources

Our research leverages data from multiple cohorts and consortiums to investigate the links between GM and OC. The primary GM data analyzed in our study were obtained from the MiBioGen consortium (https://mibiogen.gcc.rug.nl). This consortium provides a comprehensive database that includes 16S rRNA gene sequencing profiles and genotyping data from 18,340 participants representing diverse ancestries across 24 cohorts, with 78% of the participants being of European descent. Then performed age-adjusted correlations Analysis, gender, technical covariates, and genetic principal components. Since this study utilized publicly available aggregate data, no additional ethics approval or consent to participate was necessary.^[15]^ Data for OC cases were extracted from the FinnGen R9 GWAS dataset of malignant neoplasm of malignant neoplasm of ovary cases and controls, which included 1025 cases and 167,189 controls. Adjustment for covariates, including sex, age, genotyping batch, and the first 10 principal components, was performed.^[16]^ In order to gain a deeper understanding of the genetic complexities of plasma metabolites, we utilized summary data from the UK Biobank (https://gwas.mrcieu.ac.uk). This data was derived from GWAS conducted on plasma metabolites and encompassed an unprecedented number of 1091 blood metabolites and 309 metabolite ratios across a cohort of 8299 European individuals.^[17]^ To ensure consistency, all participants in the study were of European descent, and a comprehensive description of the participants can be found in Table S1, Supplemental Digital Content, http://links.lww.com/MD/N881.

### 2.3. SNP selection

To ensure the reliability of our conclusions regarding the causal relationship between GM, plasma metabolites, and the risk of OC, we implemented a rigorous quality control process to select the most appropriate IVs.^[18]^ We utilized 2 thresholds to identify SNPs significantly associated with GM and plasma metabolites as IVs. We selected only those SNPs that exhibited a genome-wide significant association (*P* < 5 × 10^−8^) with the respective traits to be used as IVs. Due to the limited number of IVs, we adjusted the significance level to 1 × 10^−5^ to mitigate potential errors arising from a restricted pool of SNPs. Subsequently, we utilized linkage disequilibrium clumping to remove undesirable specific SNPs (with *r*^2^ > 0.01 and window size < 10,000 kb).^[11]^ Afterwards, we harmonized the datasets for exposure and outcome variables and removed palindromic SNPs with allele frequencies close to 0.5. These stringent criteria were employed to ensure the validity and robustness of our analyses and to reduce potential biases in our results. A comprehensive description of the IVs for different categories of GM can be found in Table S2, Supplemental Digital Content, http://links.lww.com/MD/N881.

In order to assess the reliability of the genetic tool used for exposure, we calculated the F-statistic using the formula: F = *R*^2^ × [(N1 − k)/k] × (1 − *R*^2^). In this equation, *R*^2^ represents the proportion of variance explained by the selected SNPs, N represents the sample size, and k represents the number of SNPs considered. If the F-statistic in the regression analysis of SNPs on exposure is below 10, it suggests that the instrument is weak.^[19]^ Following a search on PhenoScanner V2 (http://www.phenoscanner.medschl.cam.ac.uk/),^[20]^ SNPs that were linked to confounding factors were excluded. The selected SNPs for the significant MR analysis are described in detail in Table S3, Supplemental Digital Content, http://links.lww.com/MD/N881.

### 2.4. Genetic analyses to elucidate causality

We initially conducted bidirectional MR analysis to investigate the causal relationship between GM and OC. Inverse-variance weighted (IVW) meta-analysis is an established MR method that calculates the weighted average of the Wald estimates for each selected SNP. We employed this method for the primary analysis.^[20]^ This approach provides reliable results when there is no pleiotropy within the IVs. Additionally, to account for different assumptions of horizontal pleiotropy, we utilized 4 complementary methods (MR-Egger regression,^[21]^ the weighted median (WM),^[22]^ the weighted mode and the simple mode^[23]^ to assess causal relationships). Bayesian weighted Mendelian randomization (BWMR) incorporates uncertainty in weak effects and considers pleiotropy. Causal inference is conducted using the variational expectation maximization (VEM) algorithm, and the findings obtained from BWMR are also reliable.^[24]^ To ensure the robustness of the findings, we conducted a sensitivity analysis. Cochran Q statistic was employed to evaluate heterogeneity in the IVW model. A Q value greater than the number of instruments minus 1 suggests the presence of heterogeneity and potentially invalid IVs. A *P*-value < .05 indicates the possible existence of heterogeneity.^[25]^ The MR-Egger intercept and the MR-pleiotropy residual sum and outlier (MR-PRESSO) global test were utilized to identify potential horizontal pleiotropy between IVs and the outcome.^[21,26,27]^ To mitigate the impact of individual SNPs, we performed a leave-one-out analysis to assess the robustness of the findings. Additionally, the asymmetry of the funnel plot was examined to evaluate the reliability of the results.^[27]^ Reverse MR analysis was conducted only if all MR methods supported the association between GM and OC.

### 2.5. Mediation analyses link “GM–plasma metabolites–OC”

We employed a two-step MR approach to disentangle the direct and indirect effects of GM and plasma metabolites on OC.^[28]^ Using the two-sample MR method, we first assessed the causal effect of GM on 1400 metabolites (b1) that are related to OC, and then examined the relationship between these metabolites and OC (b2). The mediation effect represents the causal influence of GM on OC through the intermediate variables. This can be estimated using the coefficient product method (b1 × b2). The direct effect (b-dir) is calculated as the difference between the total effect and the mediation effect (b-dir = b-all − b1 × b2). The mediation proportion can be calculated as the “indirect effect/total effect” ratio (b1 × b2/b-all).

All statistical analyses were conducted using R software version 4.3.2. For our MR method, we utilized the “TwoSampleMR” package, which is available in R. This package facilitated the harmonization of our datasets and enabled the implementation of various MR methods, ensuring robust and consistent results. Additionally, we employed an R-based plotting library to generate visual representations of our findings.

## 3. Result

### 3.1. Two-sample MR analysis between GM and OC

**Figure 2.**
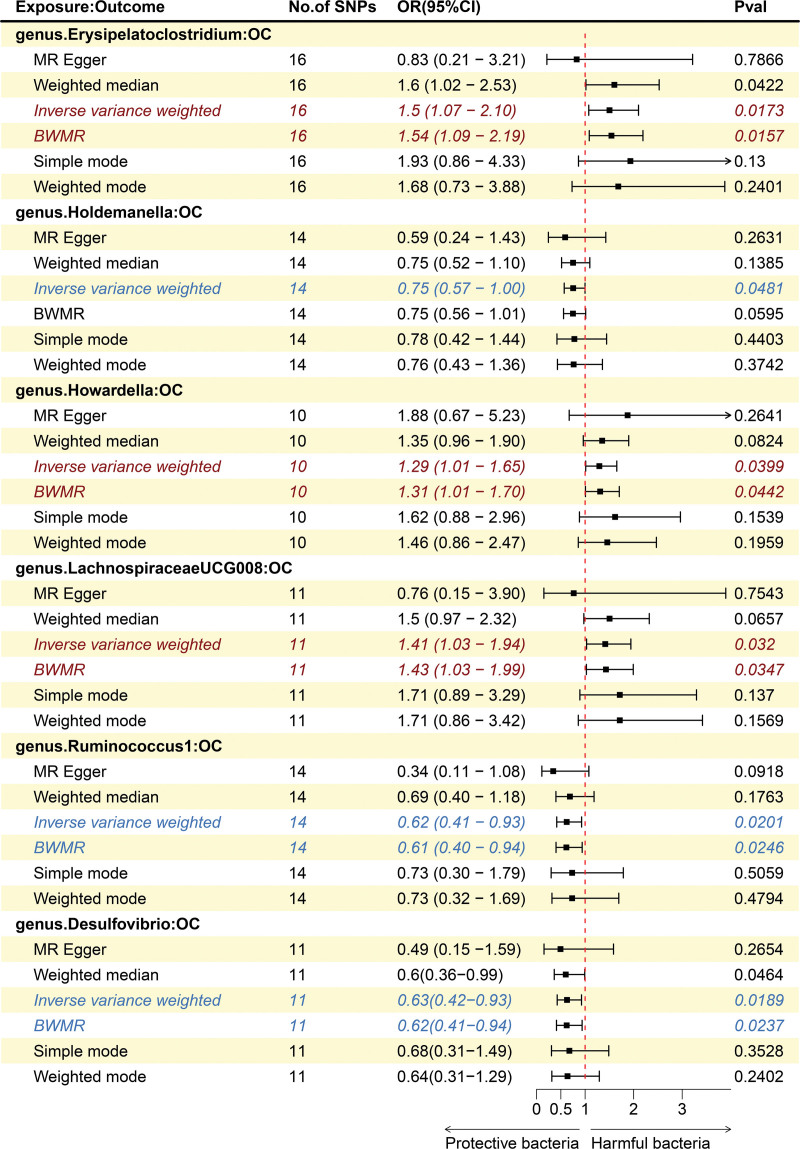
The causal effect of 6 gut microbiota on OC. The black dots represent the estimates using MR analysis, and the black bars represent the 95% confidence intervals of estimates. The OR > 1 indicates increased risk (marked in red) while < 1 indicates decreased risk (marked in blue). * Results with *P*-value < .05 were considered as suggestive associations. BWMR = Bayesian weighted Mendelian randomization; OC = ovarian cancer; OR = odds ratio; SNP = single nucleotide polymorphism.

After removing 5 unknown name bacterial groups from the 221 GM flora, we delved into the associations between 196 GM taxa (9 phyla, 16 class, 20 orders, 32 families, 113 Genera) and OC. Using the IVW method, we found suggestive evidence for a causal association between genetically predicted increases in *Erysipelatoclostridium* (odds ratio (OR) = 1.5; 95% confidence interval [CI]: 1.07–2.10; *P* = .0173), *Howardella* (OR = 1.29; 95% CI: 1.01–1.65; *P* = .0399), and *Lachnospiraceae UCG008* (OR = 1.41; 95% CI: 1.03–1.94; *P* = .032) had higher risk of OC, while genetically predicted increases in *Holdemanella* (OR = 0.75; 95% CI: 0.57–1.00; *P* = .048), *Ruminococcus1* (OR = 0.62; 95% CI: 0.41–0.93; *P* = .0201), and *Desulfovibrio* (OR = 0.63; 95% CI: 0.42–0.93; *P* = .0189) appeared to confer protective effects against OC (Fig. 2). However, the BWMR method suggested that *Holdemanella* has no significant causal relationship with OC (*P* = .0595). At the same time, MR-PRESSO (*P*-value for global test > .05), Cochran Q test (*P*-value > .05), and MR-Egger test (*P*-value > .05) are also shown IVs lack heterogeneity and horizontal pleiotropy, these results were deemed reliable (Table S4, Supplemental Digital Content, http://links.lww.com/MD/N881). The results of “leave-one-out” analysis proved that MR analysis turned out to be robustness. (The null line is not within the total confidence interval of the SNPs, Figure S1, Supplemental Digital Content, http://links.lww.com/MD/N882). The relatively symmetrical funnel plot showed that the reliability of the results (Figure S2, Supplemental Digital Content, http://links.lww.com/MD/N882). The scatter plots showed the overall effect of GM on OC (Figure S3, Supplemental Digital Content, http://links.lww.com/MD/N882). In addition, the forest plots indicated the causal associations between GM and OC (Figure S4, Supplemental Digital Content, http://links.lww.com/MD/N882).

**Figure 3.**
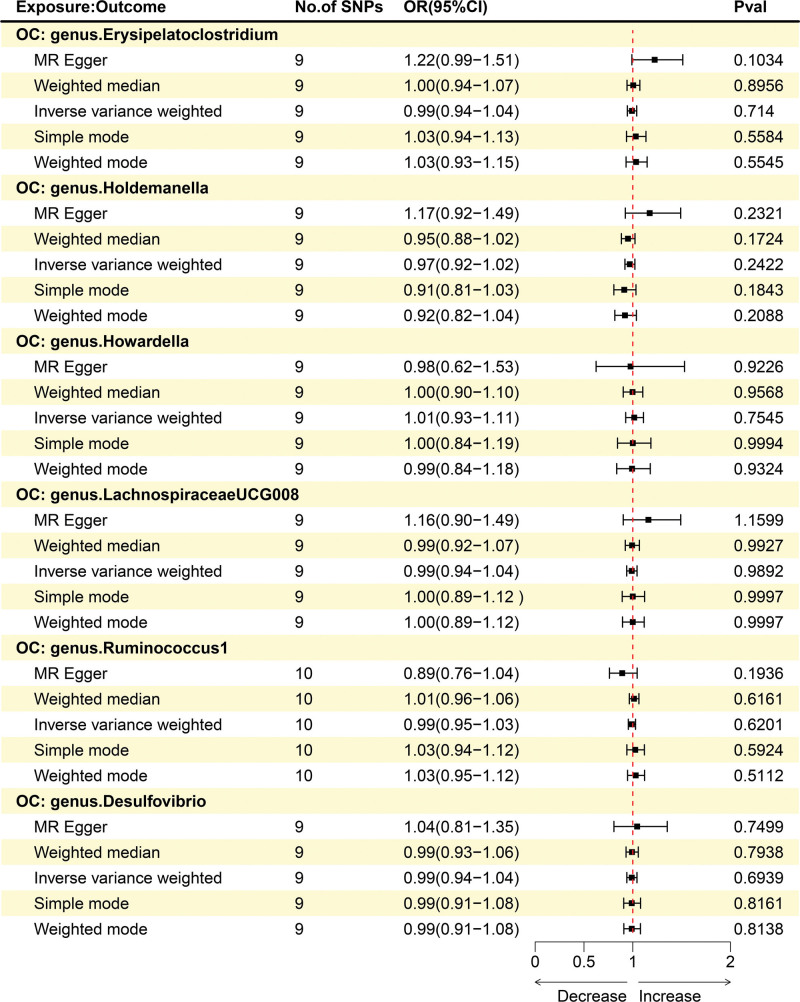
The causal effect of OC on 6 gut microbiota. OC = ovarian cancer.

Then we evaluated the potential reverse associations of these 6 bacterial traits and OC using the reverse MR analyses. As shown in Figure 3, we did not find statistically significant associations between OC and any of these 6 bacterial traits using IVW method (OR = 0.99; 95% CI: 0.94–1.04; *P* = .714 for *Erysipelatoclostridium*; OR = 0.97; 95% CI: 0.92–1.02; *P* = .2422 for *Holdemanella*; OR = 1.01; 95% CI: 0.93–1.11; *P* = .7545 for *Howardella*; OR = 0.99; 95% CI: 0.94–1.04; *P* = .9892 for *LachnospiraceaeUCG008*; OR = 0.99; 95% CI: 0.95–1.03; *P* = .6201 for *Ruminococcus 1* and OR = 0.99; 95% CI: 0.94–1.04; *P* = .6939 for *Desulfovibrio*). It has been demonstrated that there is no significant causal effect of OC on these 6 bacterial traits, which allows us to proceed with the subsequent mediation MR study. However, due to the BWMR analysis suggesting no apparent causal relationship between *Holdemanella* and OC, we will focus on studying the remaining 5 bacterial traits.

### 3.2. Mediation analyses of potential plasma metabolites

**Figure 4.**
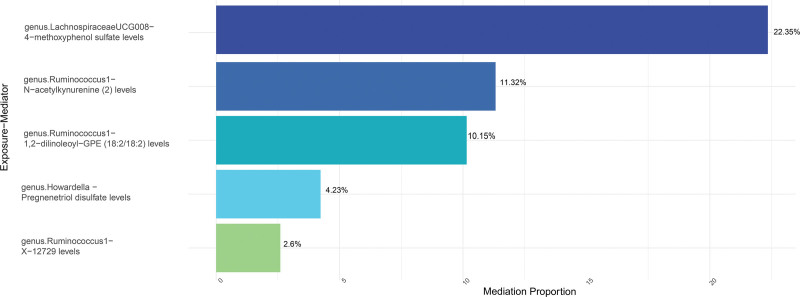
Mediation analysis of plasma metabolites between gut microbiota and OC. OC = ovarian cancer.

MR analysis was conducted on the 5 GM and 1400 plasma metabolites mentioned above. The IVW results indicated a causal relationship between these 5 GM and 305 plasma metabolites (Table S5, Supplemental Digital Content, http://links.lww.com/MD/N881 for details). Further analysis of these 305 plasma metabolites using MR analysis of metabolites and OC led to the identification of 11 plasma metabolites that may have causal relationships with OC (Table S6, Supplemental Digital Content, http://links.lww.com/MD/N881). Among the 5 GM that are causally associated with OC, 3 of them may influence OC through the aforementioned 5 metabolites. *Ruminococcus1*, a protective taxon against OC (OR = 0.62; 95% CI: 0.41–0.93; *P* = .0201), reduces the levels of 1,2-dilinoleoyl-GPE (18:2/18:2) and N-acetylkynurenine (2), both of which are risk factors for OC (OR = 1.30; 95% CI: 1.06–1.58; *P* = .0093 for 1,2-dilinoleoyl-GPE (18:2/18:2) levels and OR = 1.28; 95% CI: 1.10–1.48; *P* = .0014 for N-acetylkynurenine (2) levels). Therefore, increased levels of 1,2-dilinoleoyl-GPE (18:2/18:2) and N-acetylkynurenine (2) may have a protective effect on OC. Additionally, *Ruminococcus1* increased the levels of X-12729, which may have a protective effect on OC (OR = 0.94; 95% CI: 0.90–1.00; *P* = .0093).*Howardella* and *Lachnospiraceae UCG008*, as risk flora of OC, lead to the occurrence of OC by down-regulating pregnenetriol disulfate levels and 4-methoxyphenol sulfate levels respectively (OR = 0.88; 95% CI: 0.79–0.98; *P* = .0226 for pregnenetriol disulfate levels and OR = 0.71; 95% CI: 0.54–0.93; *P* = .0135 for 4-methoxyphenol sulfate levels). Sensitivity analysis demonstrates that these results are considered reliable (Table S7, Supplemental Digital Content, http://links.lww.com/MD/N881 and Figures S5–S14, Supplemental Digital Content, http://links.lww.com/MD/N882). As shown in Table 1 and Figure 4, we calculated the indirect effects and meditation proportion by these metabolites, and we observed an indirect effect of 4-methoxyphenol sulfate levels between *Lachnospiraceae UCG008* and OC, with the meditation proportion of 22.35%; N-acetylkynurenine (2) levels, the meditation proportion of 1,2-dilinoleoyl-GPE (18:2/18:2) levels and X-12729 levels between *Ruminococcus 1* and OC were 11.32%, 10.15%, and 2.6%, respectively; pregnenetriol disulfate levels mediated between *Howardella* and OC was 4.23%.

**Table 1:** Two-step Mendelian randomization analysis of causal relationships between gut microbiota, plasma metabolites, and OC.

Mediator	Outcome	Total effect (b-all)	Direct effect	Mediation effect (b1 × b2)	Mediation proportion
Pregnenetriol disulfate levels	OC	0.256959276	0.246092513	0.010866763	4.23%
4-Methoxyphenol sulfate levels	OC	0.346853338	0.269339903	0.077513435	22.35%
1,2-Dilinoleoyl-GPE (18:2/18:2) levels	OC	-0.477283982	-0.428855656	-0.048428326	10.15%
N-acetylkynurenine (2) levels	OC	-0.477283982	-0.423265928	-0.054018054	11.32%
X-12729 levels	OC	-0.477283982	-0.464870298	-0.012413684	2.60%

Total effect (b-all): the effect of GM on the OC risk.

Direct effect β1(b1): the effect of the GM on the plasma metabolites. Direct effect β2(b2) the effect of the plasma metabolites on the OC risk.

Mediation effect: the effect of GM on the OC risk acting through the plasma metabolites.

GM = gut microbiota, OC = ovarian cancer.

## 4. Discussion

In the era of precision medicine, working knowledge of microbial communities may play a role in understanding whether modulation of the GM affects treatment patterns in clinical practice and represents a new and important adjunct to current anticancer strategies. The GM is considered a potential modulator of the risk of multiple malignancies, including OC, through interactions with metabolites and/or the host immune system.^[5,29]^ Our study investigates the relationship between specific taxa of GM and OC by employing MR, which offers valuable insights into this intricate interaction. In the present large-scale MR study, 6 GM taxa were causally associated with OC. The *genus Erysipelatoclostridium*, genus *Howardella*, and genus *LachnospiraceaeUCG008* have been found to be positively associated with OC. This suggests that an increased abundance of these taxa might be linked to a higher risk of developing OC. The genus *Holdemanella*, genus *Ruminococcus1*, and genus *Desulfovibrio* show a negative association with OC. This indicates that these taxa might have a protective effect against the disease.

Microorganisms affect tumorigenesis by altering key cellular pathways. This can happen through direct interactions between microbial proteins and human cell surface receptors or by binding metabolites produced by microbes.^[30]^ Bacterial pathogens like *Helicobacter pylori*, *S enterica*, and *F nucleatum* use surface proteins (CagA and AvrA) to attach to human cell receptors. This interaction activates signaling pathways, such as β-catenin and STAT3, resulting in inflammation, DNA damage, or premalignant changes.^[30–33]^ *F nucleatum* produces Fap2 lectins that specifically bind to the Gal-GalNAc moiety in tumors like ovarian adenocarcinoma. Recent studies show a connection between *F nucleatum* and OC with high Gal-GalNAc levels indicating a preference for these tumors.^[30,34]^ Microorganisms produce toxins and metabolites in the host’s circulatory system that can affect cancer progression, such as the CagA protein from *H pylori*, which is associated with tumor development.^[35]^ A healthy estrogen-gut axis is crucial for estrogen metabolism and utilization, while dysbiosis of the estrobolome is indirectly linked to ovarian carcinoma.^[36]^ These findings underscore the significance of GM and metabolites in cancer. However, much of the existing literature relies on case–control studies that identify associations with specific diseases but do not establish causation.

Using two-steps MR analysis, we found that some plasma metabolites can participate in the effects of microbiota on OC. We suggest that genus *LachnospiraceaeUCG008* may increase the risk of OC by reducing the levels of 4-methoxyphenyl sulfate. Genus *Ruminococcus1* may exert a protective effect on OC patients through 1,2-dilinoleoyl-GPE (18:2/18:2), N-acetylcanavanine (2), and X-12729. Genus *Howardella* may increase the risk of OC by reducing the levels of pregnenetriol disulfate.

*Lachnospiraceae* is a significant family of anaerobic bacteria within the phylum *Firmicutes*, closely related to *Ruminococcus*. These bacteria ferment indigestible dietary fibers to produce beneficial fatty acids, such as butyrate, which contribute to energy provision and maintenance of intestinal barrier integrity. However, certain members of the *Lachnospiraceae* family have been linked to aging and metabolic disorders, indicating a complex role in human health.^[37]^ The significant mediation proportion, particularly for genus *Lachnospiraceae UCG008* and genus *Ruminococcus 1*, suggests that the metabolites produced by these microbiota taxa may play a crucial role in influencing OC.

4-Methoxyphenol sulfate, a tyrosine metabolite important in cancer control, has demonstrated antitumor properties.^[37,38]^ Previous study demonstrated that 4-methoxyphenol sulfate inhibited tumor growth in a preclinical model of azoxymethane (AOM)-induced colon carcinogenesis.^[39]^ In line with prior studies, the findings suggest that the presence of the *Lachnospiraceae UCG008* genus may play a role in the development of OC through the reduction of serum concentration of 4-methoxyphenol sulfate.

More than 90% of dietary tryptophan is metabolized through the kynurenine pathway.^[40]^ Indoleamine 2,3-dioxygenase (IDO) and tryptophan 2,3-dioxygenase (TDO) serve as the initial and rate-limiting enzymes in the kynurenine pathway. Overactivation of the kynurenine pathway, particularly IDO, is associated with an unfavorable prognosis in various cancer types, including gastrointestinal cancers, gynecological cancers, hematological malignancies, breast cancer, lung cancer, glioma, melanoma, prostate cancer, and pancreatic cancer. Furthermore, kynurenine promotes cancer cell invasion, metastasis, and resistance to chemotherapy.^[41]^ Studies have demonstrated that elevated IDO expression was detected in 56.7% of patients with OC, and this was correlated with reduced overall survival and progression-free survival.^[42]^ Kynurenine induces T regulatory cell differentiation, leading to enhanced production of anti-inflammatory cytokines and suppression of T cell cytotoxic activity. Excessive activation of the kynurenine pathway in cancer creates an immunosuppressive microenvironment that facilitates the survival and invasion of mutant cells into surrounding tissues.^[41]^ Experimental evidence indicates that N-acetylkynurenine (NAK) has biological activity similar to kynurenine. It regulates activated macrophages via the aryl hydrocarbon receptor (AhR), which, when activated, suppresses inflammation by limiting pro-inflammatory cytokine secretion and enhancing immunoregulatory mediators. NAK may inhibit IL-6 transcription by activating the AhR pathway and reducing promoter activity. Furthermore, NAK can lower IL-6 production by suppressing histamine release in LPS-stimulated macrophages, thus inhibiting inflammatory responses.^[43]^ Our study further confirmed the positive causal relationship between NAK and OC. Interestingly, studies have confirmed a significant increase in the abundance of *Ruminococcus* in patients with elevated intestinal oxidative stress.^[44]^ We hypothesize that *Ruminococcus* may play a promotive role in the inflammatory response due to its abundance in the body. Consequently, it might inhibit the levels of NAK and subsequently impede OC development. However, there are currently no relevant studies showing that 1,2-dilinoleoyl-GPE (18:2/18:2) may be related to the disease, and further exploration is needed in the future.

Pregnenetriol disulfate is a metabolite of steroids. There is currently very little research on Pregnenetriol disulfate. Only 1 study has shown a significant increase in pregnenetriol disulfate levels in patients with COVID-19, suggesting that changes in steroid profiles may contribute to oxidative stress.^[45]^ We hypothesized for the first time that the genus *Howardella* may decrease the level of pregnenetriol disulfate in serum, potentially contributing to the onset and progression of OC by inhibiting oxidative stress in the body. Research on the genus. *Howardella* in tumors is limited. Currently, only 1 MR study suggested a potential negative correlation between *Howardella* and small intestinal cancer.^[46]^ However, our results are inconsistent with this finding. Further experiments are required to investigate this phenomenon.

This study possesses several strengths: Firstly, this study is the first to assess the causal relationship among GM–plasma metabolites–OC. Secondly, our study distinguishes itself through a comprehensive approach that integrates rigorous analyses. The consistent findings across methods, including BWMR and primary IVW, enhance the robustness of our conclusions. The utilization of the MR-PRESSO strategy enhances the credibility of our findings by identifying and correcting potential outliers, thereby reducing bias. Thirdly, the homogeneity of our study sample, predominantly of European ancestry, minimizes bias arising from population variations.

Nevertheless, our study has limitations. There are many factors that lead to OC. We have focused on GM and their metabolites in the development of OC but have overlooked the spontaneous parthenogenetic activation of ovarian oocytes that may lead to ovarian tumors including teratomas.^[47]^Furthermore, the other limitation arises from the substantial reliance on data from the European population, potentially introducing biases and restricting the generalizability of our results to other ethnic groups. Moreover, the absence of individual-level data restricts our investigation of intricate associations, possibly neglecting nonlinear relationships among GM, plasma metabolite levels, and OC. Consequently, specific association patterns, such as U-shaped or J-shaped relationships, may be disregarded. Potential nonlinear and interactive effects between GM and OC need to be carefully considered in future studies.

## 5. Conclusion

To our knowledge, this study represents the first comprehensive assessment of the causal relationship between GM, plasma metabolites, and OC. These findings underscore the significance of elucidating the underlying mechanisms connecting GM and OC. Moreover, these results offer novel insights into microbiome-based therapies and targeted interventions focused on metabolites for the treatment of OC.

## Author contributions

**Conceptualization:** Yu Wang, Yangyu Liu, Xinyue Liu.

**Data curation:** Yangyu Liu, Yongai Li, Xinyue Liu.

**Formal analysis:** Shanxiang Gao, Yan Han.

**Funding acquisition:** Xinyue Liu.

**Investigation:** Yu Wang, Shanxiang Gao, Yongai Li, Xinyue Liu.

**Methodology:** Yangyu Liu, Hui Yao, Yan Han.

**Project administration:** Hui Yao.

**Resources:** Hui Yao.

**Software:** Shanxiang Gao, Yangyu Liu, Yan Han.

**Supervision:** Yu Wang,Yongai Li.

**Validation:** Yu Wang,Yongai Li.

**Visualization:** Shanxiang Gao.

**Writing – original draft:** Xinyue Liu, Yu Wang

**Writing – review & editing:** Yongai Li, Xinyue Liu.

## Supplementary Material


