# Supplementary material for: Association between gut microbiota, plasma metabolites, and ovarian cancer: A Mendelian randomization study
Source: Medicine (Baltimore). 2024 Nov 8;103(45):e40479. doi: 10.1097/MD.0000000000040479 (PMC11556969; doi:10.1097/MD.0000000000040479)
Supplement: Supplementary file 2 [file medi-103-e40479-s002.pdf]

Supplementary Figures legends

Supplementary Figure 1. MR leave-one-out sensitivity analysis for Gut microbiota on OC.

A. Analysis for " genus.Erysipelatoclostridium " on "OC"

B. Analysis for " genus.Holdemanella" on "OC"

C. Analysis for " genus.Howardella " on "OC"

D. Analysis for " genus.LachnospiraceaeUCG008 " on "OC"

E. Analysis for " genus.Ruminococcus1 " on "OC"

F. Analysis for " genus.Desulfovibrio " on "OC"

A

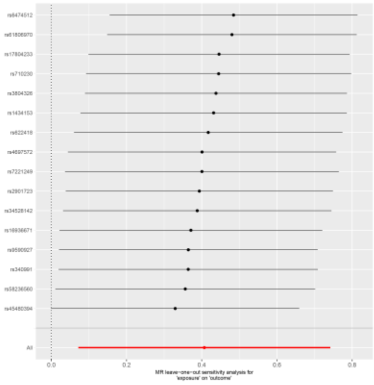

genus.Erysipelatoclostridium

B

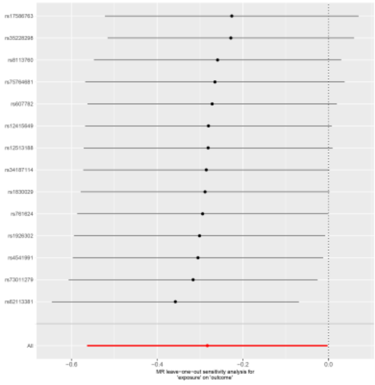

genus.Holdemanella

C

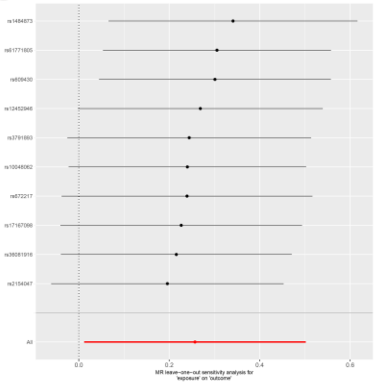

genus.Howardella

D

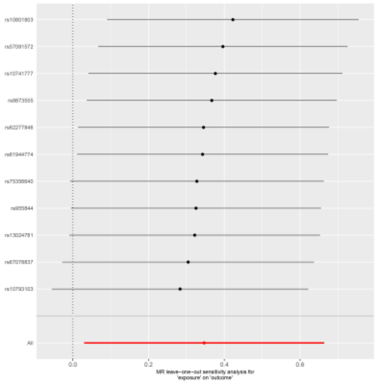

genus.LachnospiraceaeUCG008

E

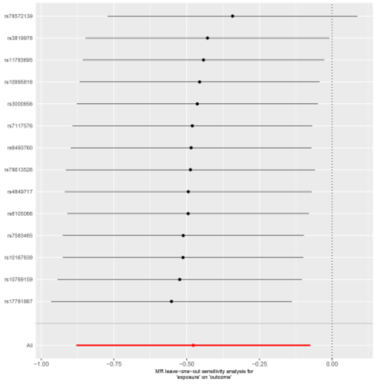

genus.Ruminococcus1

F

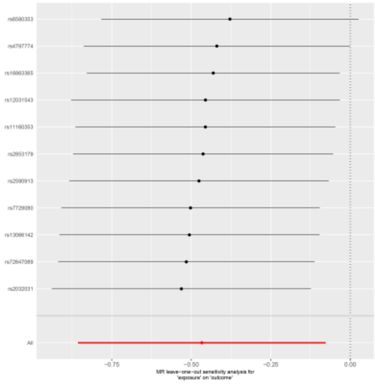

genus.Desulfovibrio

Supplementary Figure 2. Funnel plots for the effect of Gut microbiota on OC.

A. Analysis for " genus.Erysipelatoclostridium " on "OC"

B. Analysis for " genus.Holdemanella" on "OC"

C. Analysis for " genus.Howardella " on "OC"

D. Analysis for " genus.LachnospiraceaeUCG008 " on "OC"

E. Analysis for " genus.Ruminococcus1 " on "OC"

F. Analysis for " genus.Desulfovibrio " on "OC"

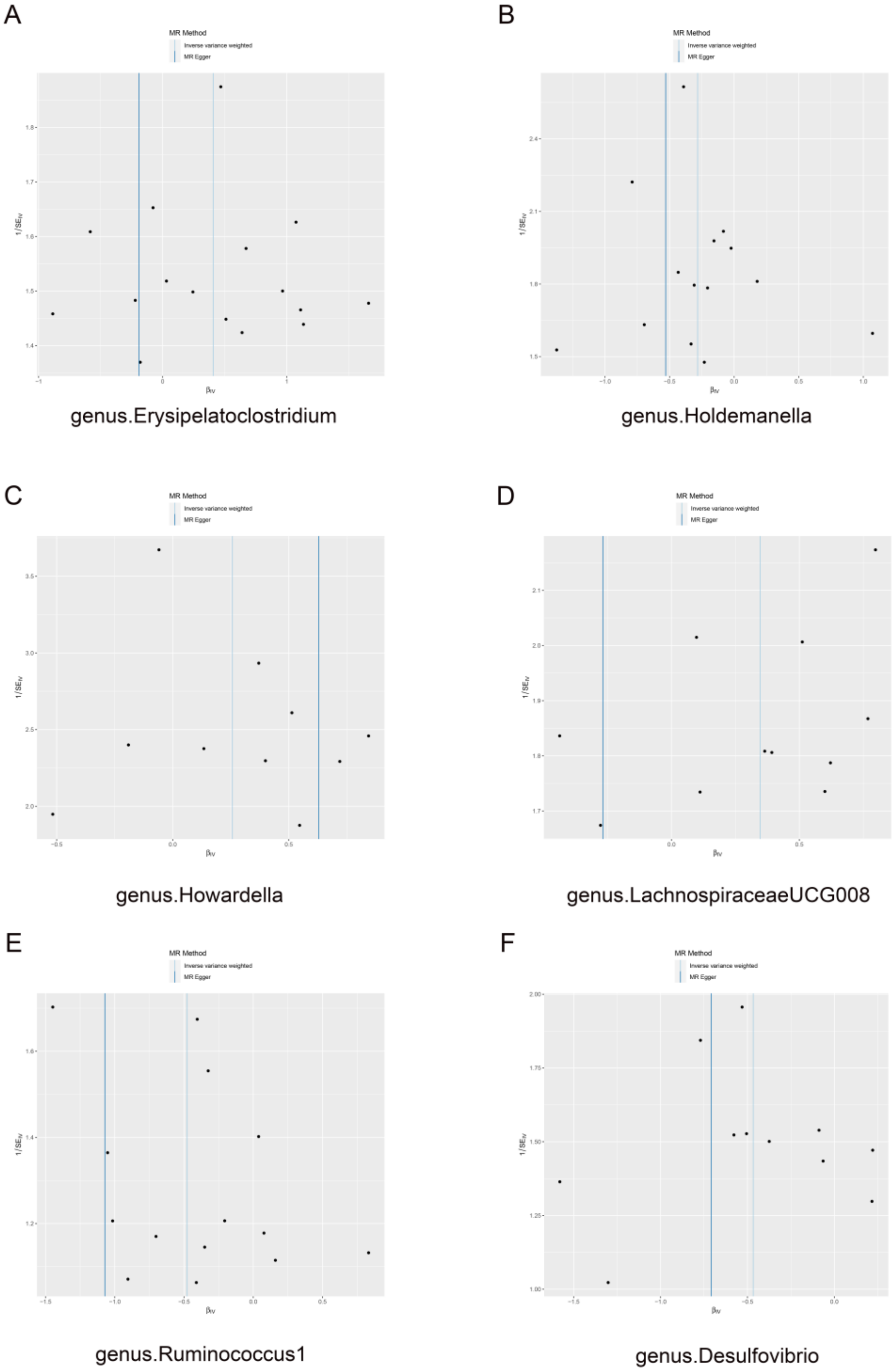
A
MR Method
Inverse variance weighted
MR Egger
genus.Erysipelatoclostridium
B
genus.Holdemanella
C
genus.Howardella
D
genus.LachnospiraceaeUCG008
E
genus.Ruminococcus1
F
genus.Desulfovibrio

Supplementary Figure 3. Scatter plots for the effect of Gut microbiota on OC.

A. SNP effect on " genus.Erysipelatoclostridium "

B. SNP effect on " genus.Holdemanella"

C. SNP effect on " genus.Howardella "

D. SNP effect on " genus.LachnospiraceaeUCG008 "

E. SNP effect on " genus.Ruminococcus1 "

F. SNP effect on " genus.Desulfovibrio "

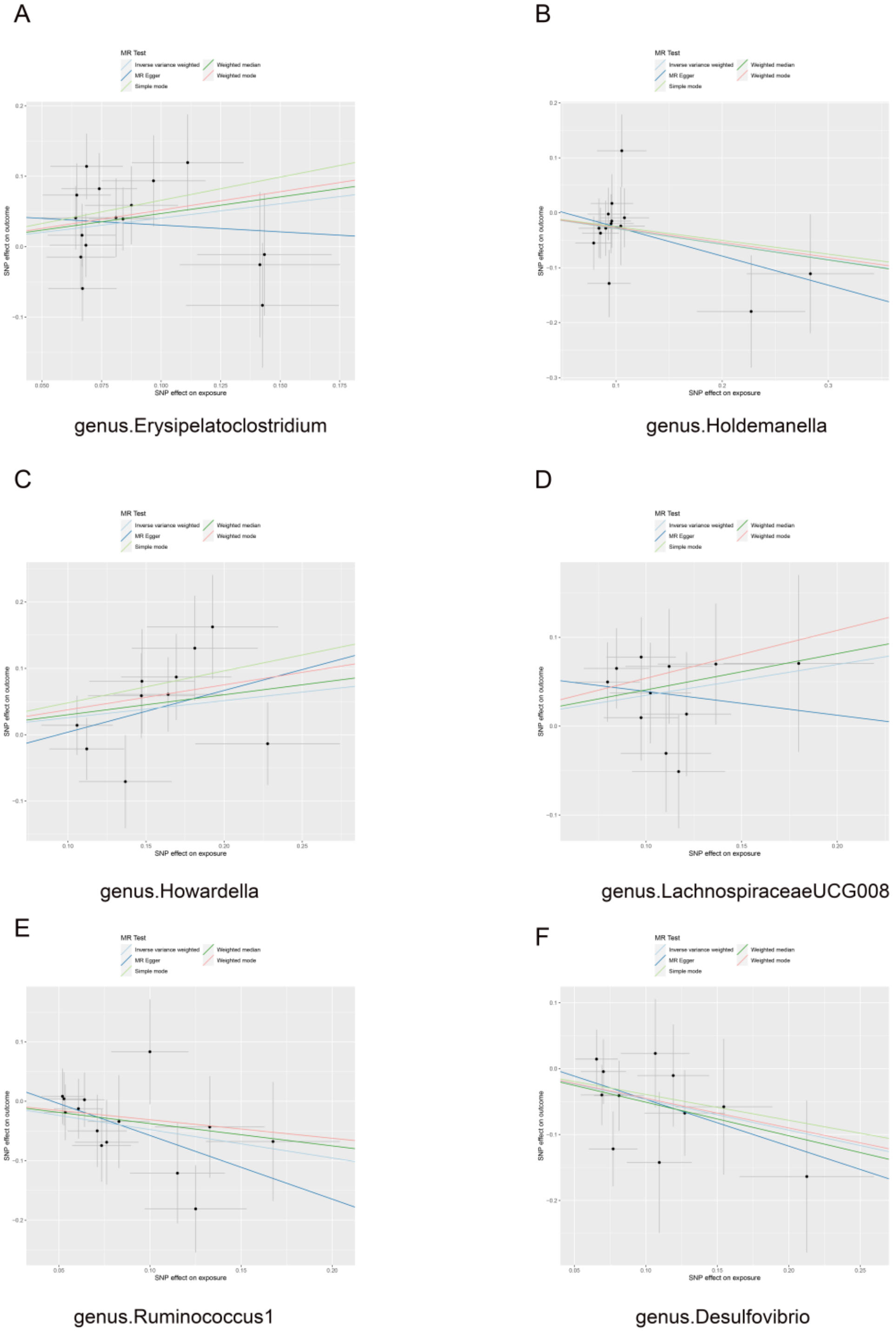
A
B
C
D
E
F
MR Test
Inverse variance weighted
MR Egger
Simple mode
Weighted median
Weighted mode
SNP effect on outcome
SNP effect on exposure
genus.Erysipelatoclostridium
genus.Holdemanella
genus.Howardella
genus.LachnospiraceaeUCG008
genus.Ruminococcus1
genus.Desulfovibrio

Supplementary Figure 4. Forest plots for the effect of Gut microbiota on OC.

A. MR effect size for " genus.Erysipelatoclostridium " on "OC"

B. MR effect size for " genus.Holdemanella" on "OC"

C. MR effect size for " genus.Howardella " on "OC"

D. MR effect size for " genus.LachnospiraceaeUCG008 " on "OC"

E. MR effect size for " genus.Ruminococcus1 " on "OC"

F. MR effect size for " genus.Desulfovibrio" on "OC"

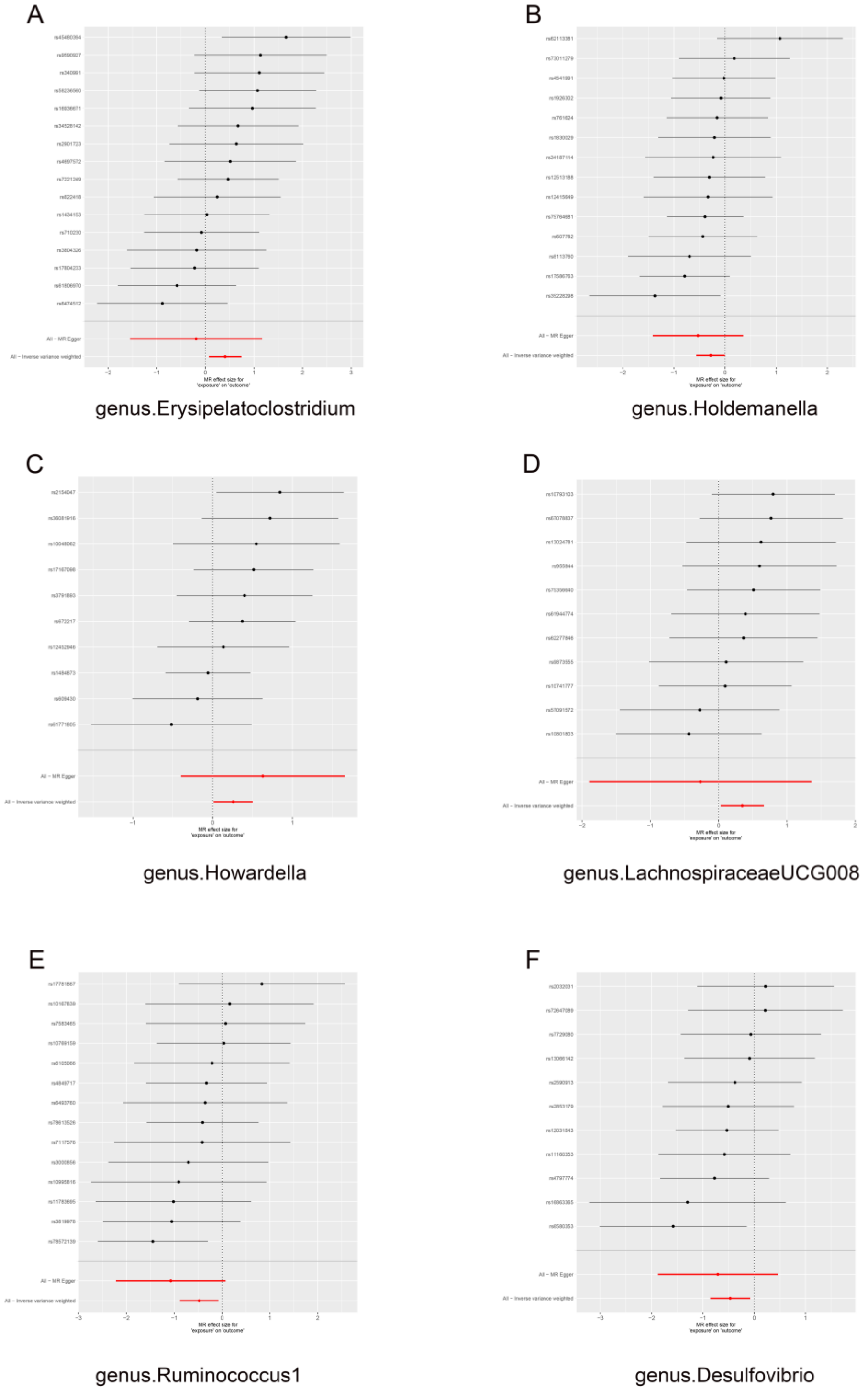
A
genus.Erysipelatoclostridium
B
genus.Holdemanella
C
genus.Howardella
D
genus.LachnospiraceaeUCG008
E
genus.Ruminococcus1
F
genus.Desulfovibrio

Supplementary Figure 5. MR leave-one-out sensitivity analysis, Funnel plots ,Scatter plots and Forest plots for Ruminococcus1 on 1,2-dilinoleoyl-GPE (18:2/18:2) levels.

A. MR leave-one-out sensitivity analysis for " genus.Ruminococcus1 " on "1,2-dilinoleoyl-GPE (18:2/18:2) levels "

B. Funnel plots for the effect of " genus.Ruminococcus1 " on "1,2-dilinoleoyl-GPE (18:2/18:2) levels "

C. Scatter plots for the effect of" genus.Ruminococcus1 " on "1,2-dilinoleoyl-GPE (18:2/18:2) levels "

D. Forest plots of MR effect size for" genus.Ruminococcus1 " on "1,2-dilinoleoyl-GPE (18:2/18:2) levels "

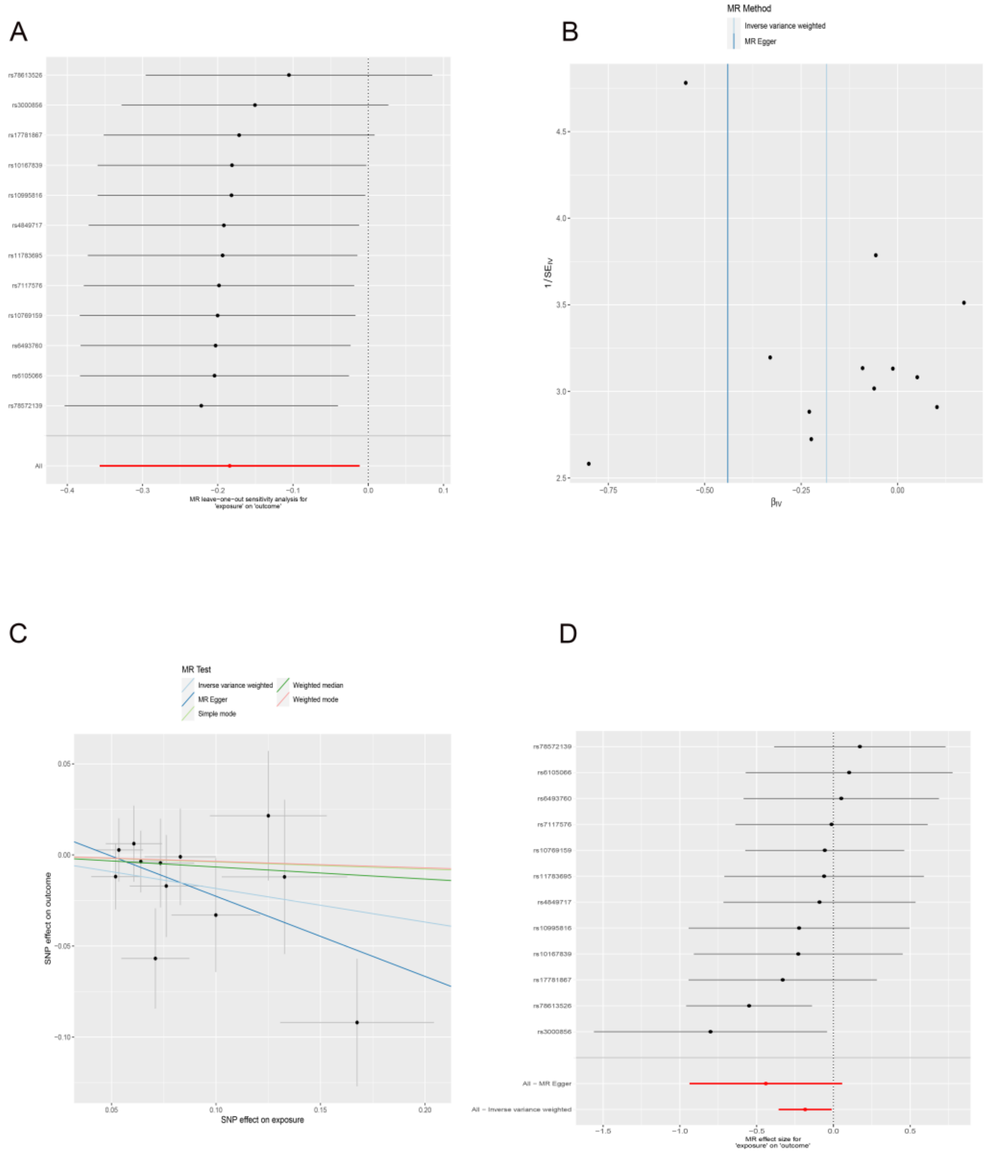
A
rs78613526
rs3000856
rs17781867
rs10167839
rs10995816
rs4849717
rs11783695
rs7117576
rs10769159
rs6493760
rs6105066
rs78572139
All
−0.4
−0.3
−0.2
−0.1
0.0
0.1
MR leave−one−out sensitivity analysis for 'exposure' on 'outcome'
B
MR Method
Inverse variance weighted
MR Egger
1/SE_IV
β_IV
C
MR Test
Inverse variance weighted
MR Egger
Simple mode
Weighted median
Weighted mode
SNP effect on outcome
SNP effect on exposure
D
rs78572139
rs6105066
rs6493760
rs7117576
rs10769159
rs11783695
rs4849717
rs10995816
rs10167839
rs17781867
rs78613526
rs3000856
All − MR Egger
All − Inverse variance weighted
MR effect size for 'exposure' on 'outcome'

Supplementary Figure 6. MR leave-one-out sensitivity analysis, Funnel plots ,Scatter plots and Forest plots for Ruminococcus1 on N-acetylkynurenine (2) levels.

A. MR leave-one-out sensitivity analysis for " genus.Ruminococcus1 " on " N-acetylkynurenine (2) levels "

B. Funnel plots for the effect of " genus.Ruminococcus1 " on " N-acetylkynurenine (2) levels "

C. Scatter plots for the effect of" genus.Ruminococcus1 " on " N-acetylkynurenine (2) levels "

D. Forest plots of MR effect size for" genus.Ruminococcus1 " on " N-acetylkynurenine (2) levels "

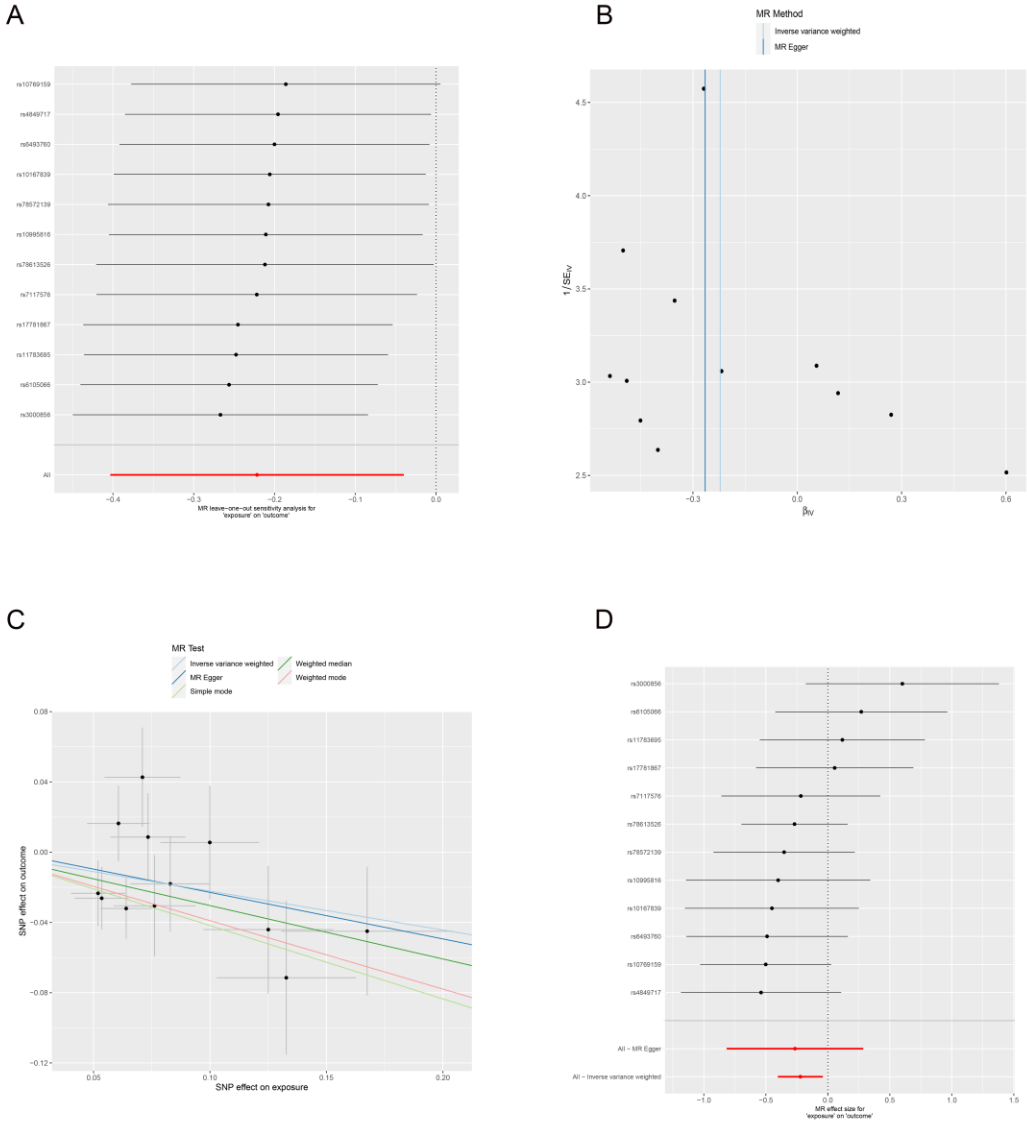
A
rs10769159
rs4849717
rs6493760
rs10167839
rs78572139
rs10995816
rs78613526
rs7117576
rs17781867
rs11783695
rs6105066
rs3000856
All
−0.4 −0.3 −0.2 −0.1 0.0
MR leave−one−out sensitivity analysis for
'exposure' on 'outcome'
B
MR Method
Inverse variance weighted
MR Egger
1 / SE_IV
4.5 4.0 3.5 3.0 2.5
−0.3 0.0 0.3 0.6
β_IV
C
MR Test
Inverse variance weighted
MR Egger
Simple mode
Weighted median
Weighted mode
SNP effect on outcome
0.08 0.04 0.00 −0.04 −0.08 −0.12
0.05 0.10 0.15 0.20
SNP effect on exposure
D
rs3000856
rs6105066
rs11783695
rs17781867
rs7117576
rs78613526
rs78572139
rs10995816
rs10167839
rs6493760
rs10769159
rs4849717
All − MR Egger
All − Inverse variance weighted
−1.0 −0.5 0.0 0.5 1.0 1.5
MR effect size for
'exposure' on 'outcome'

Supplementary Figure 7. MR leave-one-out sensitivity analysis, Funnel plots ,Scatter plots and Forest plots for Ruminococcus1 on X-12729 levels.

A. MR leave-one-out sensitivity analysis for " genus.Ruminococcus1 " on " X-12729 levels "

B. Funnel plots for the effect of " genus.Ruminococcus1 " on " X-12729 levels "

C. Scatter plots for the effect of" genus.Ruminococcus1 " on " X-12729 levels "

D. Forest plots of MR effect size for" genus.Ruminococcus1 " on " X-12729 levels "

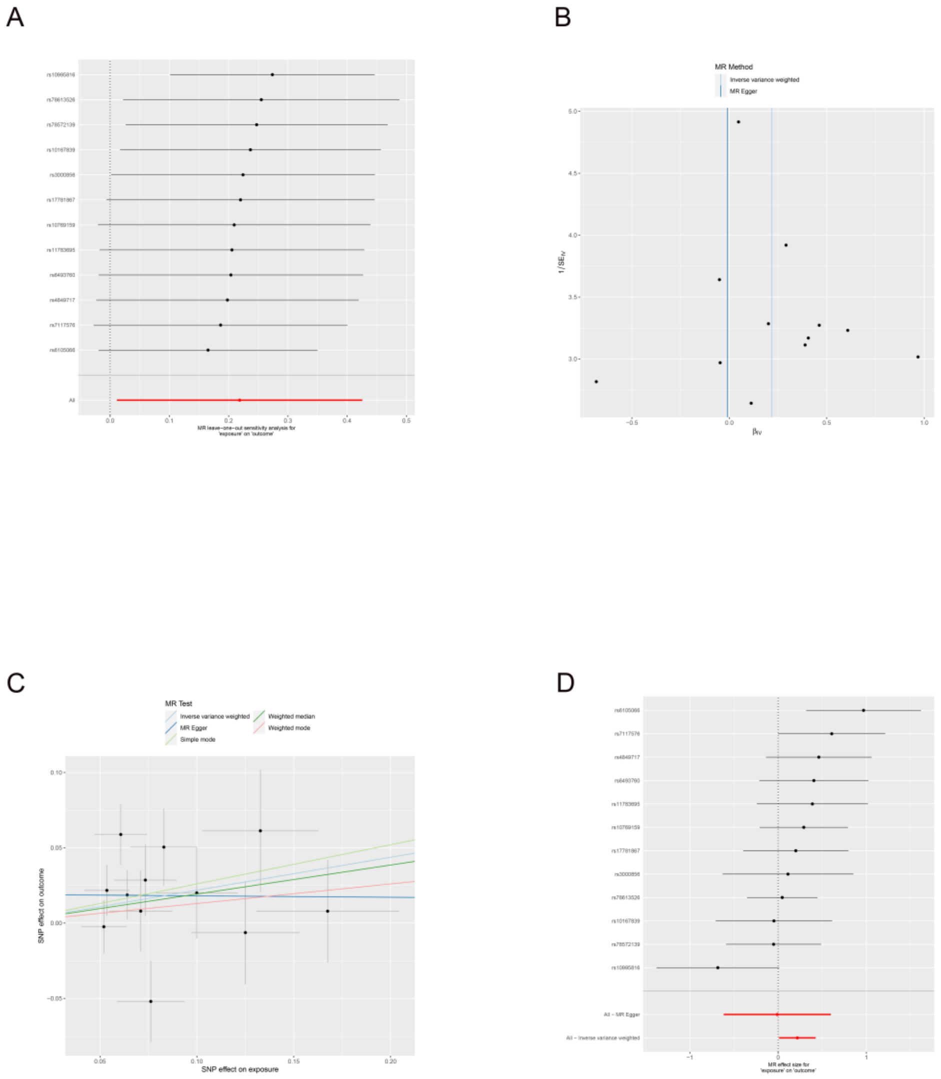
A
B
MR Method
Inverse variance weighted
MR Egger
C
MR Test
Inverse variance weighted
MR Egger
Simple mode
Weighted median
Weighted mode
SNP effect on outcome
SNP effect on exposure
D

Supplementary Figure 8. MR leave-one-out sensitivity analysis, Funnel plots, Scatter plots and Forest plots for Howardella on Pregnenetriol disulfate levels.

A. MR leave-one-out sensitivity analysis for " genus. Howardella " on " Pregnenetriol disulfate levels "

B. Funnel plots for the effect of " genus. Howardella " on " Pregnenetriol disulfate levels "

C. Scatter plots for the effect of" genus. Howardella " on " Pregnenetriol disulfate levels "

D. Forest plots of MR effect size for" genus. Howardella " on " Pregnenetriol disulfate levels "

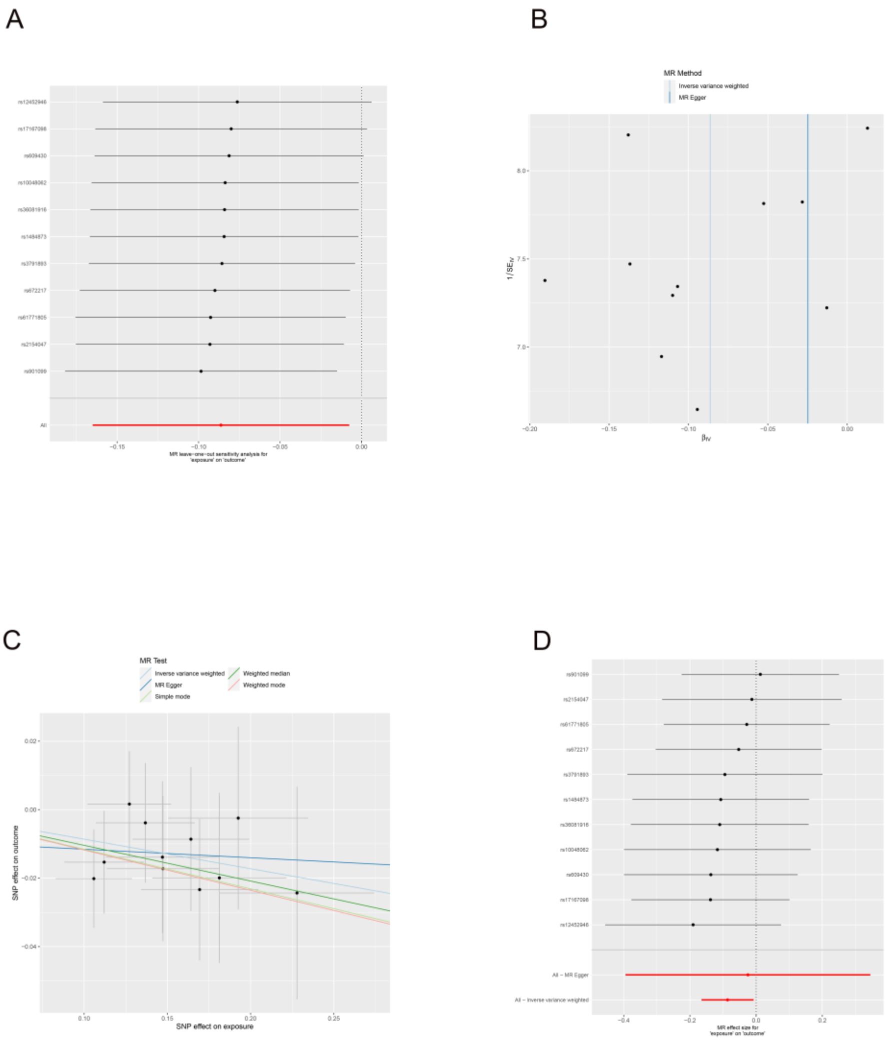
A
B
MR Method
Inverse variance weighted
MR Egger
C
MR Test
Inverse variance weighted
Weighted median
MR Egger
Weighted mode
Simple mode
SNP effect on outcome
SNP effect on exposure
D

Supplementary Figure 9. MR leave-one-out sensitivity analysis, Funnel plots, Scatter plots and Forest plots for LachnospiraceaeUCG008 on 4-methoxyphenol sulfate levels.

A.MR leave-one-out sensitivity analysis for " genus. LachnospiraceaeUCG008 " on " 4-methoxyphenol sulfate levels"

B. Funnel plots for the effect of " genus. LachnospiraceaeUCG008 " on " 4-methoxyphenol sulfate levels"

C. Scatter plots for the effect of " genus. LachnospiraceaeUCG008 " on " 4-methoxyphenol sulfate levels"

D. Forest plots of MR effect size for " genus. LachnospiraceaeUCG008 " on " 4-methoxyphenol sulfate levels"

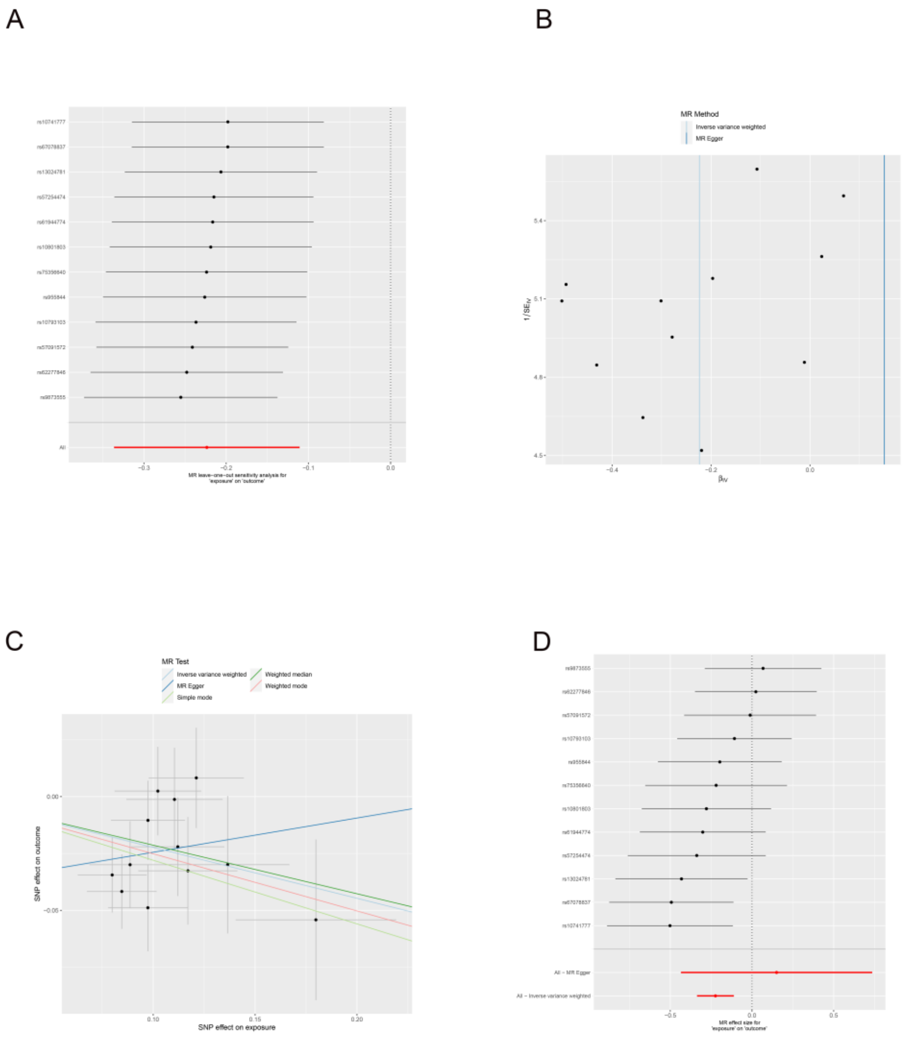
A
B
C
D
MR leave−one−out sensitivity analysis for 'exposure' on 'outcome'
MR Method
Inverse variance weighted
MR Egger
1/SE
MR Test
Inverse variance weighted
MR Egger
Simple mode
Weighted median
Weighted mode
SNP effect on exposure
SNP effect on outcome
All − MR Egger
All − Inverse variance weighted
MR effect size for 'exposure' on 'outcome'

Supplementary Figure 10. MR leave-one-out sensitivity analysis, Funnel plots ,Scatter plots and Forest plots for 1,2-dilinoleoyl-GPE (18:2/18:2) levels on OC.

A. MR leave-one-out sensitivity analysis for " 1,2-dilinoleoyl-GPE (18:2/18:2) levels " on "OC"

B. Funnel plots for the effect of " 1,2-dilinoleoyl-GPE (18:2/18:2) levels " on "OC"

C. Scatter plots for the effect of " 1,2-dilinoleoyl-GPE (18:2/18:2) levels " on "OC"

D. Forest plots of MR effect size for " 1,2-dilinoleoyl-GPE (18:2/18:2) levels " on "OC"

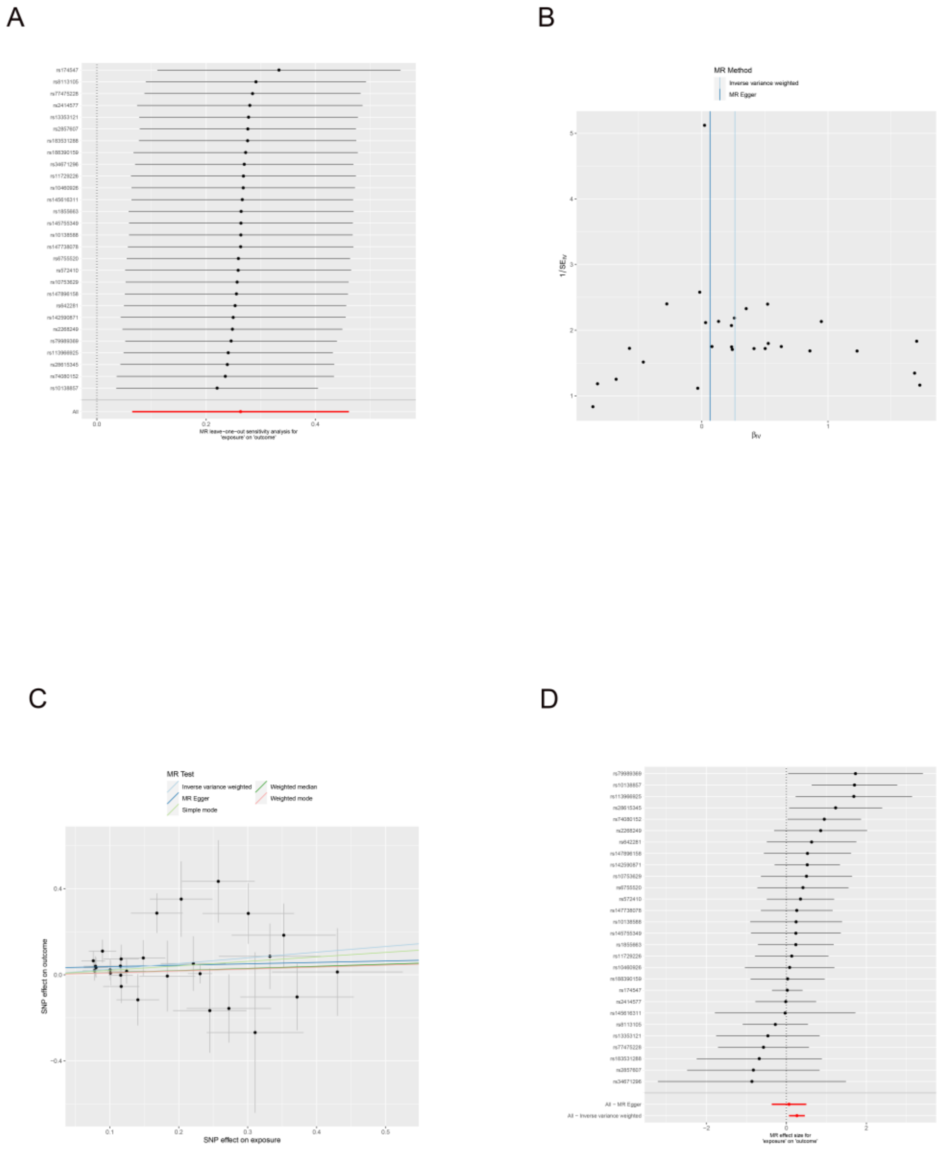
A
B
C
D
MR leave-one-out sensitivity analysis for
'exposure' on 'outcome'
MR Method
Inverse variance weighted
MR Egger
MR Test
Inverse variance weighted
MR Egger
Simple mode
Weighted median
Weighted mode
SNP effect on exposure
SNP effect on outcome
MR effect size for
'exposure' on 'outcome'
All - MR Egger
All - Inverse variance weighted

Supplementary Figure 11. MR leave-one-out sensitivity analysis, Funnel plots ,Scatter plots and Forest plots for N-acetylkynurenine (2) levels on OC.

A. MR leave-one-out sensitivity analysis for " N-acetylkynurenine (2) levels " on "OC"

B. Funnel plots for the effect of " N-acetylkynurenine (2) levels " on "OC"

C. Scatter plots for the effect of " N-acetylkynurenine (2) levels " on "OC"

D. Forest plots of MR effect size for " N-acetylkynurenine (2) levels " on "OC"

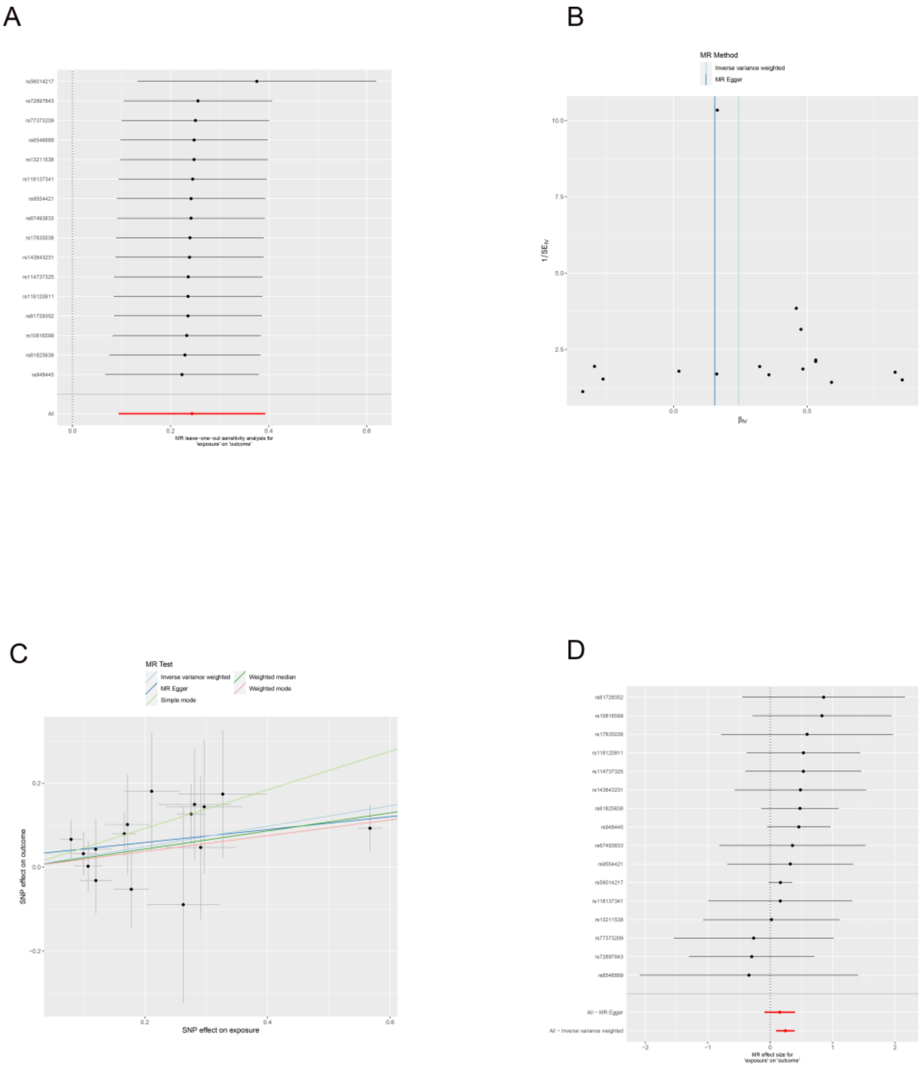
A
All
MR leave-one-out sensitivity analysis for
'exposure' on 'outcome'
B
MR Method
Inverse variance weighted
MR Egger
C
MR Test
Inverse variance weighted
MR Egger
Simple mode
Weighted median
Weighted mode
SNP effect on outcome
SNP effect on exposure
D
All − MR Egger
All − Inverse variance weighted
MR effect size for
'exposure' on 'outcome'

Supplementary Figure 12. MR leave-one-out sensitivity analysis, Funnel plots ,Scatter plots and Forest plots for X-12729 levels on OC.

A. MR leave-one-out sensitivity analysis for " X-12729 levels " on "OC"

B. Funnel plots for the effect of " X-12729 levels " on "OC"

C. Scatter plots for the effect of " X-12729 levels " on "OC"

D. Forest plots of MR effect size for " X-12729 levels " on "OC"

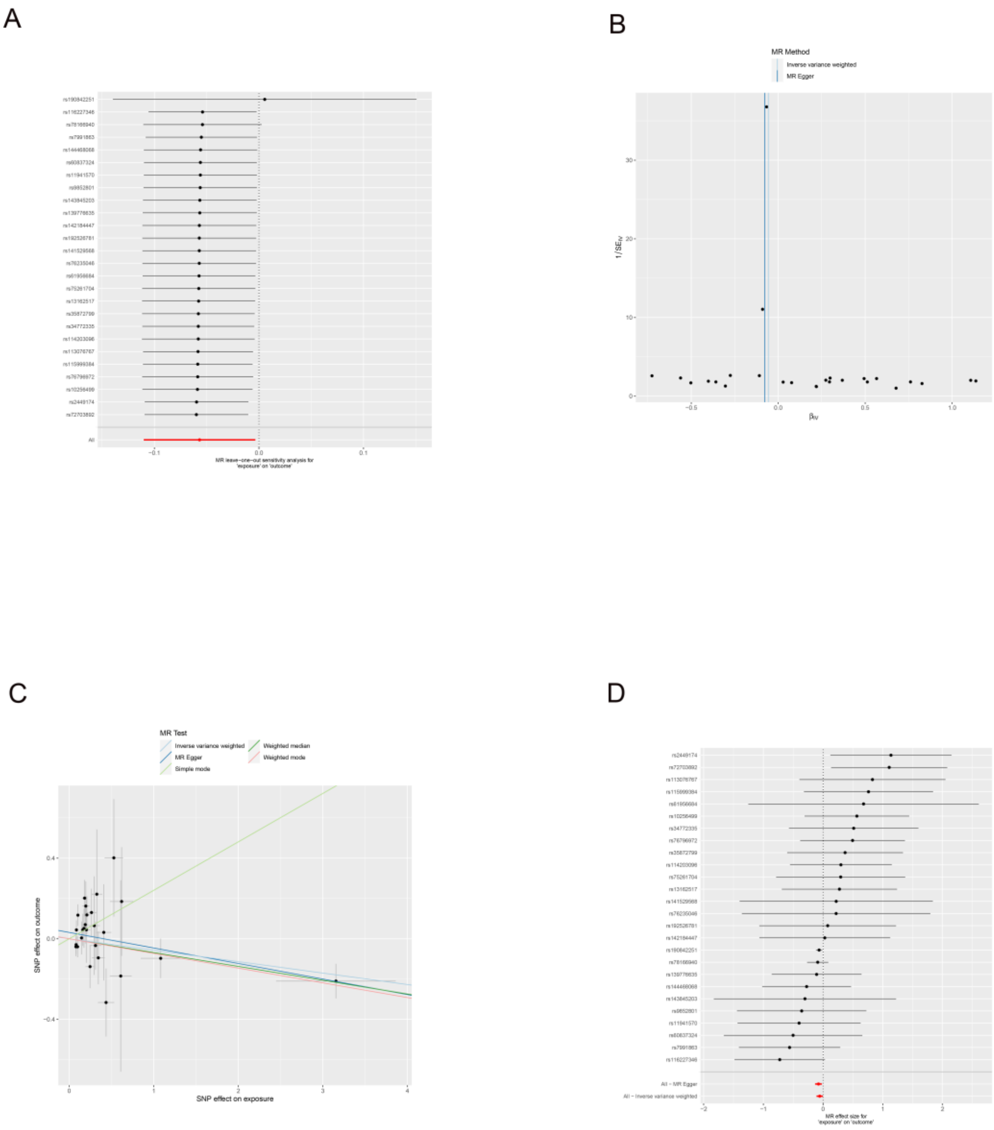
A
B
MR Method
Inverse variance weighted
MR Egger
C
MR Test
Inverse variance weighted
MR Egger
Simple mode
Weighted median
Weighted mode
SNP effect on outcome
SNP effect on exposure
D

Supplementary Figure 13. MR leave-one-out sensitivity analysis, Funnel plots ,Scatter plots and Forest plots for Pregnenetriol disulfate levels on OC.

A. MR leave-one-out sensitivity analysis for " Pregnenetriol disulfate levels " on "OC"

B. Funnel plots for the effect of " Pregnenetriol disulfate levels " on "OC"

C. Scatter plots for the effect of " Pregnenetriol disulfate levels " on "OC"

D. Forest plots of MR effect size for" Pregnenetriol disulfate levels " on "OC"

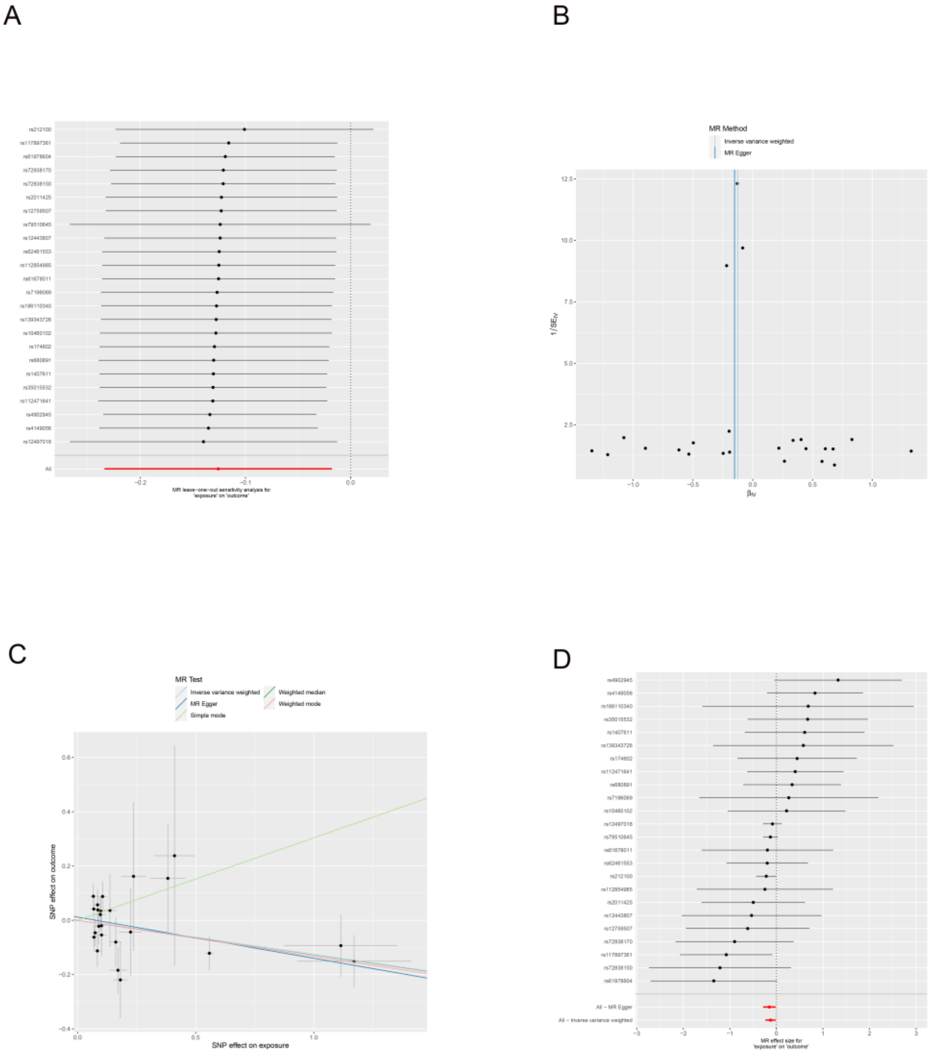
A
MR leave-one-out sensitivity analysis for 'exposure' on 'outcome'
All
B
MR Method
Inverse variance weighted
MR Egger
C
MR Test
Inverse variance weighted
MR Egger
Simple mode
Weighted median
Weighted mode
SNP effect on outcome
SNP effect on exposure
D
All – MR Egger
All – Inverse variance weighted
MR effect size for 'exposure' on 'outcome'

Supplementary Figure 14. MR leave-one-out sensitivity analysis, Funnel plots, Scatter plots and Forest plots for 4-methoxyphenol sulfate levels on OC.

A. MR leave-one-out sensitivity analysis for " 4-methoxyphenol sulfate levels " on "OC"

B. Funnel plots for the effect of " 4-methoxyphenol sulfate levels " on "OC"

C. Scatter plots for the effect of " 4-methoxyphenol sulfate levels " on "OC"

D. Forest plots of MR effect size for" 4-methoxyphenol sulfate levels " on "OC"

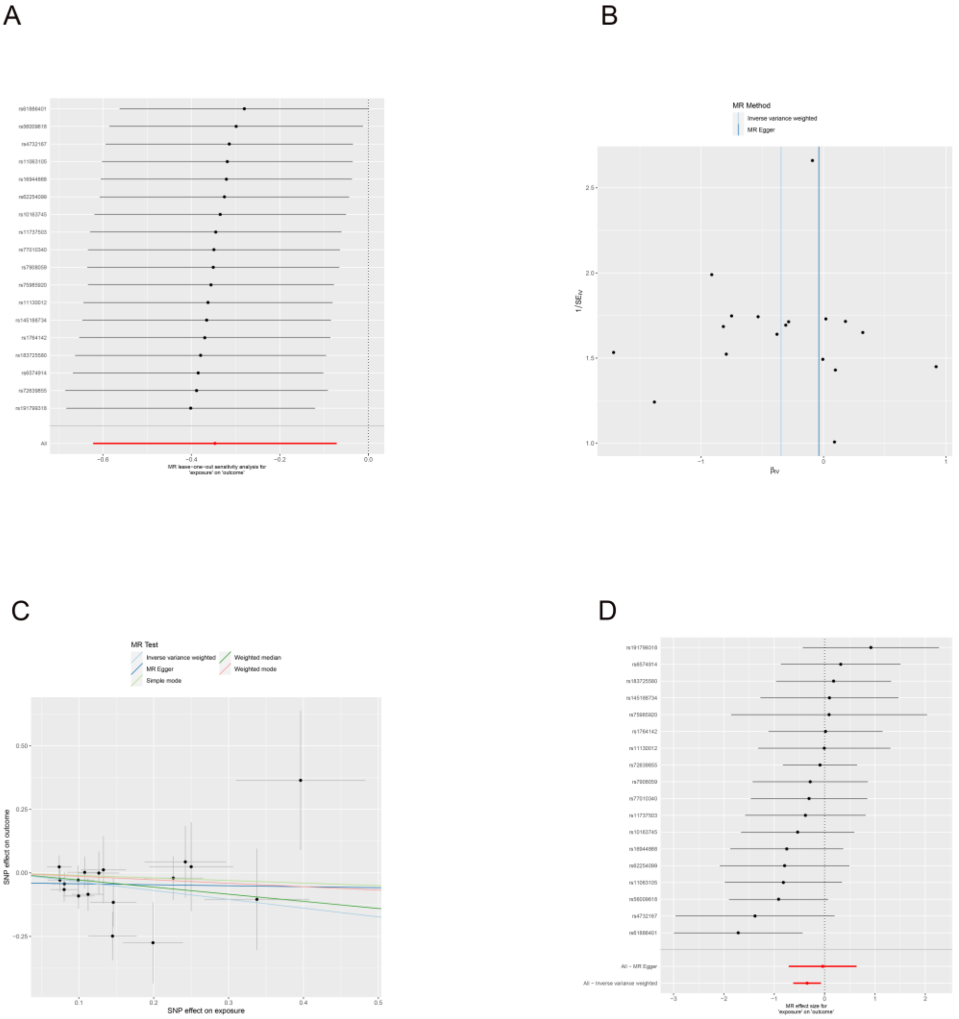
A
All
MR leave−one−out sensitivity analysis for
'exposure' on 'outcome'
B
MR Method
Inverse variance weighted
MR Egger
1/SE_IV
β_IV
C
MR Test
Inverse variance weighted
MR Egger
Simple mode
Weighted median
Weighted mode
SNP effect on outcome
SNP effect on exposure
D
All − MR Egger
All − Inverse variance weighted
MR effect size for
'exposure' on 'outcome'
